# Cellulose Extraction from Soybean Hulls and Hemp Waste by Alkaline and Acidic Treatments: An In-Depth Investigation on the Effects of the Chemical Treatments on Biomass

**DOI:** 10.3390/polym17091220

**Published:** 2025-04-29

**Authors:** Antonella Moramarco, Edoardo Ricca, Elisa Acciardo, Enzo Laurenti, Pierangiola Bracco

**Affiliations:** 1 Department of Chemistry, University of Torino, Via Pietro Giuria 7, 10125 Torino, Italy; antonella.moramarco@unito.it (A.M.); enzo.laurenti@unito.it (E.L.); 2 SUSPLAS@UniTo, Sustainable Plastic Scientific Hub, University of Torino, Via Pietro Giuria 7, 10125 Torino, Italy; 3 NIS Interdepartmental Centre, University of Torino, 10125 Torino, Italy

**Keywords:** cellulose fibers, biomass valorization, agricultural waste, soybean hulls, hemp straw, chemical characterization

## Abstract

The agri-food supply chain and other industries that convert agricultural raw materials into various consumer goods generate large quantities of by-products, most of which end up in landfills. This waste, rich in cellulose, provides a significant opportunity for the conversion of agricultural residues into valuable products. In this paper, soybean hulls and hemp waste were subjected to chemical treatments with alkaline (NaOH 2% *w*/*v*) and acidic solutions (HCl 1 M) to remove non-cellulosic components and isolate cellulose. The biomass was characterized after each chemical process through FTIR, SEM, EDX, elemental analysis, TGA, and XRD. Lignin was determined following two different procedures, a conventional TAPPI protocol and a method recently proposed in the literature (CASA method). The results indicated that the chemical treatments favored the removal of organic compounds and minerals, increasing the cellulose content in biomass after each step. The purified product of soybean hulls consists of fibers 35–50 µm long and 5–11 µm thick, containing nearly pure cellulose arranged in crystalline domains. Fibers of variable sizes, rich in crystalline cellulose, were isolated from hemp waste. These fibers have diameters ranging between 2 and 60 µm and lengths from 40 to 800 µm and contain considerable amounts of lignin (~14%).

## 1. Introduction

The limited availability of petrochemical resources and increasing environmental challenges have encouraged the transition from a linear economy to a circular one, promoting the development of new products from natural resources and post-consumer/post-industrial materials. Although the production of renewable and sustainable products has gained increasing interest in recent years, the use of these materials dates back further. The automotive sector was one of the pioneering industries that replaced conventional fillers with natural ones. In the 1950s, Trabant was launched in the German automotive market; the peculiarity of this car was related to the material used for the body shell, a mixture of polymers and cotton fibers [1].

Nowadays, plant fibers are used in several sectors, from automotive to building, since they offer several advantages, such as low cost, low density, high specific strength, high modulus, and a non-abrasive nature [2,3]. The term “plant fibers” denotes elongated vegetable cells, typically having lengths ranging from 1 to 50 mm and diameters from 10 to 50 µm [4]. They can be classified into six categories based on the tissue from which they are extracted: seed, bast, leaf, fruit, wood, and grass and reed fibers [5,6]. Most of the non-wood fibers consist of sclerenchyma cells [7], a class of cells whose morphology, from spherical to cylindrical, varies with the plant tissue (cortex, leaves, stems, etc.) [8]. Plant fibers have an internal cavity named lumen surrounded by two cell walls: the primary wall and the secondary wall. The primary wall is the outermost barrier; it is a thin layer and contains an irregular network of cellulose microfibrils. On the contrary, the secondary wall is thicker and comprises three sublayers: S1 is the outermost, S3 is the innermost, and S2 lies in the middle. S2, the thickest layer, contains highly oriented cellulose microfibrils and is the most relevant layer for the mechanical performance of plant fibers [4,9,10]. The outstanding mechanical performance of plant fibers is ascribable to the complex hierarchical structure of cellulose chains.

Cellulose is a linear homopolysaccharide containing D-glucopyranose units linked by β-1,4 glycosidic bonds. The polar groups inside the structural unit promote the formation of hydrogen bonds between consecutive units of the same chain (intramolecular bonds) and between units of different chains (intermolecular bonds). Intramolecular hydrogen bonds partially provide stiffness to the polymer, whereas intermolecular bonds promote the formation of three-dimensional structures named microfibrils [10,11]. Microfibrils typically have a diameter of 10–30 nm and are surrounded by hemicelluloses and lignin, which act as cementing materials in the fiber bundle. Microfibrils contain 30 to 100 cellulose chains arranged in crystalline and amorphous regions [4,6]. The crystalline structure of cellulose varies with the source; in plants, cellulose has mainly a triclinic symmetry (named Iβ), whereas primitive organisms show a monoclinic symmetry (named Iα) [10].

The crystalline structure provides peculiar properties to cellulose, such as insolubility in several solvents, high mechanical performance (tensile strength, axial stiffness, etc.), and thermal stability, which prevents cellulose from melting [11]. In addition to crystallinity, other parameters, e.g., cellulose degree of polymerization and microfibrillar angle, influence the performance of plants. The microfibrillar angle is defined as the angle formed between cellulose microfibrils and the fiber axis. High degrees of polymerization and low microfibrillar angles enhance the mechanical properties of plant fibers [4].

In addition to cellulose, plant fibers contain several components, such as hemicelluloses, lignin, pectins, proteins, and waxes, whose relative abundance varies with the source and geographical origin of the plant. Among all components, cellulose, hemicelluloses, and lignin have a structural function in plants.

Hemicelluloses are a heterogeneous group of polysaccharides, mainly composed of xyloglucans, xylans, mannans, glucomannans, and β-(1→3, 1→4)-glucans [12]. Hemicelluloses act as coupling agents between the hydrophilic cellulose and the hydrophobic lignin, forming hydrogen bonds with the surface groups of cellulose microfibrils, covalent bonds with lignin, and ester bonds with hydroxycinnamic acids [11,13,14,15]. The amorphous structure and free hydroxyl groups give hemicelluloses low thermal stability and high reactivity. These polysaccharides have a lower degree of polymerization than cellulose and can be hydrolyzed in acidic environments [11].

Lignin is a hydrophobic polymer and contains three phenylpropane units, guaiacyl, syringyl, and p-hydroxyphenyl, linked by aryl ether and carbon–carbon bonds forming a three-dimensional network. Lignin is soluble in hot alkali and is sensitive to oxidation, whereas it is stable in acidic solutions [11,16].

The remarkable properties of plant fibers have promoted their use as reinforcing agents in composites. However, the presence of low thermally stable components, e.g., hemicelluloses and pectins, limited their applicability to polymers processable below 200 °C [17]. The need to remove thermally unstable components from biomass has encouraged the research of sustainable methods for cellulose isolation [18,19,20]. Mechanical treatments exploit impact, shear, and hydrodynamic forces to separate micrometric fibers from biomass. Since these methods require high energy consumption, they are usually combined with chemical or enzymatic treatments, which favor biomass defibrillation [21,22]. Alkaline, acidic, and bleaching agents facilitate the removal of non-cellulosic components from biomass; hence, they are widely employed in chemical treatments. Alkaline solutions play several functions: they promote the swelling of the fibers and the partial breaking of the hydrogen bonding network, enhancing the surface of the fibers [16,23]; furthermore, some bonds connecting lignin and hemicelluloses are broken in alkaline media [14]. Lignin is sensitive to alkaline environments; the concentration of the solution and the type of base influence its solubilization [24]. During the chemical treatments, alkaline solutions with a concentration lower than 17.5 wt% are employed to prevent modifications in the cellulose structure [4,16]. Higher concentrations transform the crystalline structure of cellulose from I to II [10].

Cellulose is sensitive to acids since acidic environments promote the hydrolysis of the glycosidic bonds in polysaccharides. Concentrated mineral acids degrade the amorphous regions of cellulose fibers, causing the formation of cellulose nanocrystals; by contrast, cellulose structure is less affected by mild acidic solutions that lead to a partial depolymerization [25]. Bleaching treatments are employed to remove lignin [11]; however, some substrates containing low amounts of lignin, e.g., soybean hulls, do not require this purification step.

Soybeans are the most cultivated crop in the world, and their global production has increased over the years, exceeding 400 million tons in 2024 [26]. Proteins and oils are extracted from soybeans and employed mainly in the food industry [27]. The production of these value-added products leads to the formation of considerable amounts of hulls every year, the main by-products of the soybean industry. The hulls, corresponding to 6–8% of soybean weight, are rich in cellulose (29–51%) and hemicelluloses (10–25%), whereas they contain low amounts of proteins, pectins, fats, lignin, and inorganics [19]. Soybean hulls, accounting for 23–32 million tons, are employed as animal feed; however, large amounts are still left to waste [28]. The large availability of soybean hulls and their composition make them a promising source of cellulose fibers.

Hemp is one of the oldest crops for textile production and is nowadays cultivated worldwide for its outstanding benefits to the environment and its multiple uses. Industrial hemp is a fast-growing crop with carbon storage and soil regeneration abilities; it requires low resources, e.g., fertilizers and pesticides, and yields more fibers per acre than other crops. Furthermore, it suppresses weeds and breaks the cycle of diseases when used in crop rotation [5]. More than 30 countries are involved in its cultivation; according to FAO Stat (2018), the major producers of industrial hemp are Canada (555,853 ha), North Korea (21,247 ha), and France (12,900 ha) [29]. In Europe, hemp production reached 179,020 tons in 2022, with France accounting for more than 60% of the European production [30]. Industrial hemp is a crop with multiple uses, being processable in more than 25,000 goods that find application in several sectors [31]. Hemp can be used in the textile, paper, cosmetic, medical, and food industries; furthermore, it is employed in the building sector as an insulator and reinforcement [29]. Hemp fibers are the cells extracted from the stem and are used in polymer composites as a reinforcement, as these fibers are particularly long and contain high amounts of crystalline cellulose [5]. The production of hemp fibers leads to the formation of considerable amounts of by-products, such as leaves, roots, and pieces of woody core, which can be returned to the soil or used for other purposes, e.g., the extraction of carbohydrates [32]. The agricultural residue originating from hemp fiber production is a heterogeneous substrate mainly containing the plant stems. The stem consists of an external protective layer, named bark, the phloem tissue, a woody core forming the xylem, and an internal cavity named pith [5,6]. The phloem and xylem tissues form the plant vascular system and conduct liquids, minerals, and sugars in the plant. Phloem and xylem differ in the substances they transport and the types of cells that form their tissue. In particular, the phloem contains sclerenchyma fibers, whereas the xylem contains xylary fibers and vessels, both of which have higher lignin content than sclerenchyma fibers [7]. The described cells are only examples of the various cell types that form the vascular system [33]. Sclerenchyma fibers extracted from the phloem tissue of hemp are classified into primary and secondary fibers. Primary fibers are longer and thicker; they are approx. 20 mm long and 10–40 µm thick, whereas secondary fibers have a length of 2 mm and a diameter of approx. 15 µm. Hemp fibers contain 53–91% cellulose, 4–18% hemicelluloses, 1–17% pectins, and 1–21% lignin. The chemical composition is influenced by several factors, e.g., cultivar, growing stage, and part of the plant from which the fibers were extracted [5].

In this paper, soybean hulls and waste obtained from hemp processing were subjected to alkaline and acidic treatments to extract cellulose fibers. There are several papers in the literature concerning the isolation of cellulose through chemical and mechanical treatments [2,34,35,36]. However, these papers mainly focus on the composition variations after the treatments and/or the final product performance. To our knowledge, there is a limited discussion about the properties of cellulose-based biomass after each chemical treatment. In this paper, we used FTIR, SEM, EDX, elemental analysis, TGA, XRD, and UV-Vis to evaluate both the effectiveness of the chemical treatments and their influence on the properties of cellulose-based biomass.

## 2. Materials and Methods

Cellulose fibers, NaOH, fuming HCl (37%), sulfuric acid (96%), and L-cysteine were purchased from Sigma Aldrich (Darmstadt, Germany) and used without further purification. Soybean hulls (particle size approx. 30 to 500 μm) and hemp waste (10 to 700 μm) were supplied in powder form by local companies and used as received.

Soybean hulls and hemp waste were chemically treated using a procedure modified from that described by Sinclair et al. [36]. The selection of reagent concentrations and treatment durations was optimized in a previous study [37], based on earlier literature findings [2,22,36]. More in detail, approx. 30 g of biomass were added to 300 mL of a NaOH solution 2% *w*/*v* and stirred at 80 °C for 2 h, then the biomass was washed with distilled water until neutrality, vacuum filtered, and air-dried. After the alkaline soaking, the biomass was subjected to acidic treatment with HCl 1 M, using the reaction conditions previously described (80 °C, 2 h, 1 g of biomass/10 mL solution) and the same purification procedure.

Alkaline treatments were alternated to acidic ones; in particular, soybean hulls were subjected to three treatments (2 alkaline, 1 acidic), whereas five treatments (3 alkaline, 2 acidic) were performed on hemp waste. The samples were labelled with the step number; odd numbers represent the alkaline steps and even numbers the acidic ones. For example, soybean 3 is the biomass obtained after alkaline-acidic-alkaline treatments.

Lignin was quantified in hemp samples (hemp waste and hemp 5) by two different methods, TAPPI T 222 om-02 [38] and a protocol reported in the literature as the CASA method [39]. Before the treatments, hemp waste was Soxhlet extracted with acetone for 24 h, whereas hemp 5 was analyzed without further purification. Following the guidelines of the TAPPI procedure, a two-stage hydrolysis was performed on 2 g of biomass. During the first step, the biomass was treated for 2 h at room temperature with 40 mL of sulfuric acid 72%, then the liquid was diluted to 3% and boiled for 4 h. The acid-insoluble lignin was filtered, air-dried, and weighed; the lignin content was calculated on the wet biomass. The supernatant was properly diluted and analyzed using UV-Vis spectroscopy to quantify soluble lignin.

The CASA method consists of an acidic hydrolysis assisted by cysteine. First, a solution of cysteine/H_2_SO_4_ was prepared in a 50 mL flask, solubilizing 5 g of cysteine in sulfuric acid 72%, then 2 mL of the cysteine solution was added to 20 mg of biomass and stirred at 60 °C for 1 h. The liquid was diluted in a 100 mL flask and analyzed by UV-Vis spectroscopy. The percentage of lignin was calculated on the wet biomass.

The CASA treatment was also performed at room temperature for 1 and 2 h. However, partial solubilization of the biomass was observed; therefore, these results will not be discussed further.

FTIR spectra were recorded in ATR mode using a Perkin-Elmer Spectrum 100 (Waltham, MA, USA). In a typical experiment, 8 scans/spectrum were collected between 4000 and 650 cm^−1^ with a resolution of 4 cm^−1^.

The morphology of the fibers was investigated by Scanning Electron Microscopy (SEM), using a Tescan VEGA3 microscope (Brno, Czech Republic). The pictures were recorded using secondary electrons and accelerated at 5 keV and 30 pA. Before the analysis, the samples were coated with a layer of gold using a VacCoat DSR1 sputter coater (London, UK).

Elemental analysis was performed with a Thermo Scientific FlashEA 1112 analyzer (Waltham, MA, USA). The sample (c.a. 2.5 mg) and an adequate amount of vanadium oxide (V2O5) were loaded in a tin capsule. The analysis was carried out in triplicate. For the statistical interpretation of data, the F-test and Student’s *t*-test (*p* < 0.05) were used.

Thermogravimetric analysis (TGA) was performed with a TA Instruments Q500 thermogravimetric analyzer (New Castle, DE, USA), using a heating ramp of 10 °C/min. The sample (8–10 mg) was heated from room temperature to 700 °C under nitrogen flux and from 700 °C to 800 °C under air flux. The analyses were performed in duplicate.

Since the samples are hygroscopic and different moisture content may affect the determination of the starting degradation temperature (T95%), the weight percentages were calculated on the dry biomass using the following equation:

$$Weight\%\left(T\right)=\frac{Weight(T)}{Weight(150\;{}^{\circ}C)}*100$$

where Weight (T) is the mass of the sample at a given temperature, and Weight (150 °C) is the mass of the sample at 150 °C.

Energy Dispersive X-ray (EDX) analysis was performed with a Tescan VEGA3 microscope coupled to an Ultim Max 40 detector, Oxford Instruments, Abingdon, UK. Before the analysis, soybean hulls and soybean 1 were heated to 800 °C under air flux in a TA Instruments Q500. The obtained ash was analyzed using an accelerating voltage of 15 keV. The chemical composition is expressed as weight percentages.

X-ray diffraction (XRD) patterns were recorded with a PANalytical X’Pert PRO MPD diffractometer (Malvern, Worcestershire, UK) having a Cu anode. The samples were finely ground in a mortar and analyzed using a Bragg–Brentano configuration. Diffractograms were acquired in the 5° ≤ 2θ ≤ 50° interval with an acquisition step of 0.02°.

The crystallinity index (CI) of cellulosic biomass was calculated using the following equation [40,41,42]:

$$CI=\frac{I_{ac}-I_{am}}{I_{ac}}*100$$

where I_ac_ and I_am_ correspond to the intensities of the crystalline phase (peak at 22.5°) and the amorphous phase (signal at 18.5°), respectively.

UV-Vis analyses were performed with a PerkinElmer Lambda 25 spectrophotometer (Waltham, MA, USA). The spectra were recorded between 200 and 700 nm with a resolution of 1 nm, using quartz cuvettes with a path length of 1 cm. Before the analyses, the liquid was centrifuged to avoid the scattering of dispersed particles (e.g., ash).

The following parameters were employed in the Lamber–Beer law for lignin quantification [39,43]:

**Wavelength (nm)****ε (L g^−1^cm^−1^)**TAPPI soluble lignin205110CASA lignin28317.25

## 3. Results and Discussion

### 3.1. Extraction of Cellulose from Soybean Hulls

#### 3.1.1. Purification Steps and Yield Measurement

Alkaline and acidic treatments were performed on soybean hulls with the aim of isolating pure cellulose fibers. The effectiveness of the treatments was confirmed by the progressive whitening of the biomass (Figure S1) and by a considerable weight loss after each step. The yields of the chemical treatments performed on soybean hulls were calculated using the following equations:

$$recovered\;biomass\;(x)=\frac{weight\;of\;the\;biomass\;after\;the\;treatment\;(x)}{weight\;of\;the\;starting\;soybean\;hulls}*100$$

$$step\;yield\;(x)=\frac{weight\;of\;the\;biomass\;after\;the\;treatment\;(x)}{weight\;of\;the\;biomass\;before\;the\;treatment\;(x)}*100$$

where x refers to the number of the treatment. The values in Table 1 indicate that the first step was responsible for the major biomass removal (−47%), whereas a moderate weight reduction was observed in the following steps, leading to a final yield of 35% (see the second column of Table 1).

The yields in the third and fourth columns of Table 1 were determined considering the wet and dry weights, respectively, measured before and after each treatment. Table 1 highlights that the presence of moisture in the samples did not significantly affect the calculation of the yields. The purification processes were performed in triplicate to evaluate the reproducibility of alkaline and acidic treatments. The yields calculated for the trials confirmed the reproducibility of the process, with values differing by less than 2%.

#### 3.1.2. FTIR Spectroscopy

**Figure 1:**
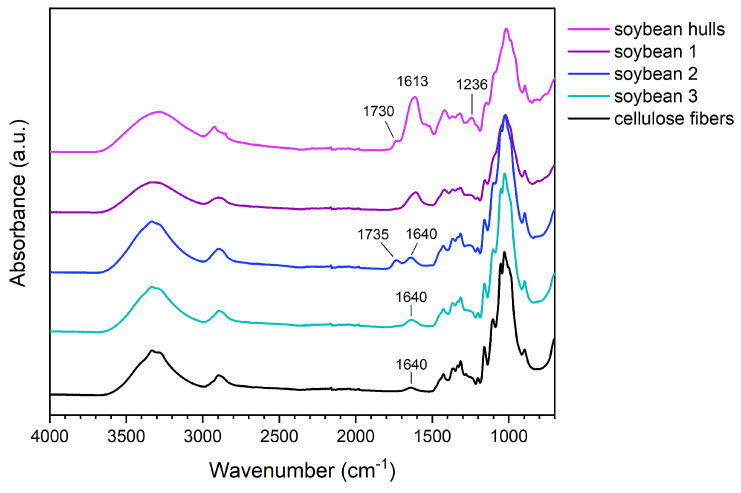
FTIR spectra of pristine cellulose and soybean hulls before and after the chemical treatments.

The effectiveness of alkaline and acidic treatments was confirmed by FTIR spectroscopy (Figure 1). In addition to cellulose signals, soybean hulls exhibit a peak at 1730 cm^−1^, a broad band centered at 1613 cm^−1^, and a peak at 1236 cm^−1^. The peak at 1730 cm^−1^, corresponding to the stretching of C=O, is attributable to the acid and ester groups of pectins [44], the pendant groups of hemicelluloses (i.e., acetyl and uronic esters), and the esters of ferulic and p-coumaric acids. The signal at 1730 cm^−1^ is also characteristic of the ester linkages connecting hemicelluloses and lignin [2,13]. In the spectrum of soybean hulls, the broad band between 1490 and 1700 cm^−1^ results from the overlapping of several signals, such as the vibrations of the amide groups of proteins and the stretching of the carboxylate groups in pectins [44] and hemicelluloses and the vibrations of the aromatic rings of lignin [45].

After the chemical treatments, variations in the biomass spectrum were observed. The spectrum of soybean 1 shows less intense signals at 1236 cm^−1^ and between 1500 and 1700 cm^−1^, suggesting that the alkaline treatment promoted a partial removal of non-cellulosic components.

It is worth noting that the peak at 1730 cm^−1^, having poor intensity in soybean 1, is visible in the spectrum of soybean 2. We hypothesize that this behavior is attributable to the non-complete removal of components having acidic groups; the first alkaline treatment promoted the dissociation of carboxylic acids and the hydrolysis of ester bonds, forming carboxylate groups; the carboxylate groups were protonated during the acidic treatment, forming carboxylic groups, which vibrate at a higher frequency than the corresponding carboxylate. The second alkaline treatment removed these components, leaving a biomass whose spectrum shows the characteristic signals of cellulose. In soybean 3, the large band between 3000 and 3700 cm^−1^ is generated by the stretching of OH groups, the signals between 2750 and 3000 cm^−1^ are characteristic of aliphatic C-H, and the weak peak at 1640 cm^−1^ corresponds to the bending of adsorbed water. Below 1500 cm^−1^, the vibrations of O-H, C-H, C-O, and C-O-C occur; methylene and alcohol groups, glycosidic bonds, and glucopyranose rings produce these signals [45,46].

The FTIR analyses highlighted that the components surrounding fiber bundles were progressively removed with the chemical treatments, leading to a progressive enrichment in cellulose. More in detail, according to the literature, the first alkaline step is fundamental in promoting both the swelling of the fibers and the disruption of hydrogen bonds. The sodium hydroxide solution converts the hydroxyl groups to alkoxide, reducing the hydrogen interactions and increasing the surface roughness; in addition, the swelling improves the exposed surface of the fibers and facilitates the diffusion of the reactants [16,47].

The alkaline environment is also responsible for the solubilization of proteins, pectins, lignin, and hemicelluloses [4,16,35,48]. Hemicellulose removal occurs both in the basic and acidic solutions; the former catalyzes the hydrolysis of the ester bonds between hemicelluloses and other components [15], and the latter promotes the hydrolysis of ether linkages in polysaccharides [2].

#### 3.1.3. Morphological Analyses

**Figure 2:**
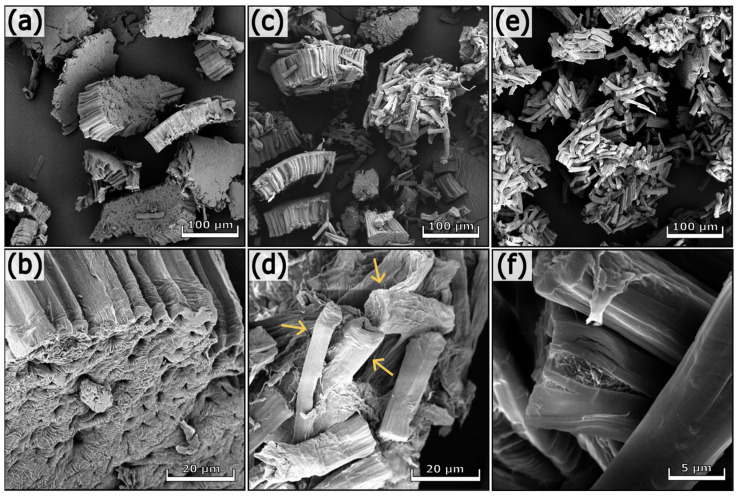
SEM images of soybean 1 (**a**,**b**), soybean 2 (**c**,**d**), and soybean 3 (**e**,**f**) at different magnifications.

The SEM images of soybean hulls before and after the treatments are illustrated in Figure S2 and Figure 2, respectively. Soybean hulls consist of different types of cells containing cellulose, hemicelluloses, and lignin in various proportions. Three main layers are detectable in soybean hulls (Figure S2); the outermost layer consists of palisade cells (PL), whereas the middle layer contains hourglass cells (HG). PL and HG belong to sclereids, dead cells with a thick secondary wall containing highly packed cellulose chains. The innermost layer contains thin-walled cells forming the parenchyma tissue (PA) [8,28,49]. As can be seen in Figure 2, the first alkaline treatment increased the superficial roughness of fibers (b), preserving the aggregation of fibers inside the bundle (a). The acidic treatment facilitated the disaggregation of fibers (c, d); however, this process was fully completed after the subsequent alkaline step (e, f). The chemical treatments removed pectins and other components that act as binders between adjacent cells and cementing material between cellulose microfibrils. As a consequence, the macroscopic organization of the fibers was destroyed, whereas the hierarchical structure of cellulose within the fibers was not significantly affected (f). It is worth noting that the final product consists of randomly oriented fibers differing in length, width, and shape (see arrows in Figure 2d). The fibers of soybean 3 have a length of 35–50 µm and a width of 5–11 µm.

#### 3.1.4. Chemical Composition

Elemental analysis (Table 2) confirmed the effectiveness of the chemical treatments. The nitrogen content, initially 1.60% in soybean hulls, decreased to 0.18% after the first alkaline treatment, indicating that nitrogen-based components, such as proteins, were removed. A significant increase in carbon and hydrogen was recorded after the acidic step, resulting in values similar to those of pristine cellulose. These results are in agreement with the literature [50].

#### 3.1.5. Thermogravimetric Analyses

The thermograms of soybean hulls and pristine cellulose are presented in Figure S3. The pyrolysis of cellulose starts at 319.8 °C (T95%) and proceeds until 400 °C, reaching the maximum speed at 348.6 °C (DTGA peak). By contrast, soybean hulls decompose through complex degradation phenomena due to the presence of components with different thermal stability. The evaporation of moisture and other volatiles occurs from room temperature up to 150 °C, whereas at higher temperatures lignocellulosic components decompose. The broad DTGA signal between 150 and 400 °C is characterized by a peak centered at 336 °C, attributable to cellulose degradation, and a shoulder at lower temperatures, generated by less stable species, such as hemicelluloses and pectins. As reported in the literature, the pyrolysis of hemicelluloses occurs approximately between 220 and 315 °C, whereas lignin degradation is observed in a wide range of temperatures, from 200 to 600 °C [20,51,52,53]. Hemicelluloses exhibit a lower thermal stability than cellulose due to their amorphous and branched structure; on the contrary, cellulose consists of packed linear chains, which provide a higher stability [52,53].

**Figure 3:**
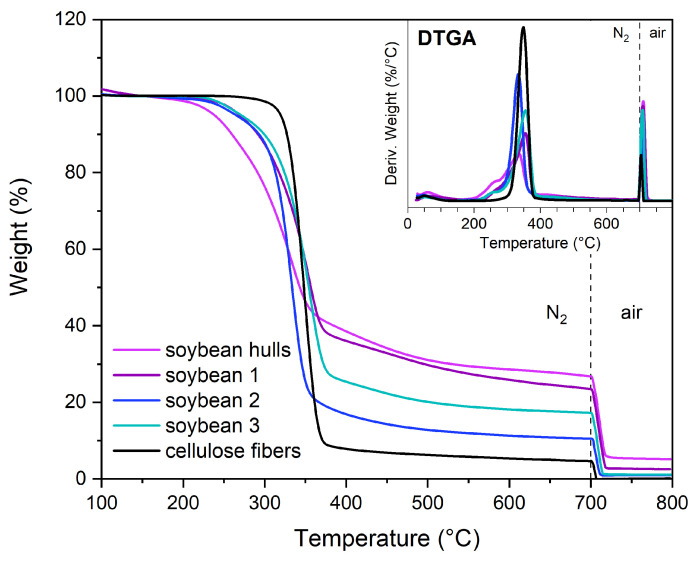
TGA and DTGA of pristine cellulose and soybean hulls before and after the chemical treatments. The dashed line represents the transition of the gas flow from nitrogen to air.

As shown in Figure 3, the chemical treatments improved the thermal stability of soybean hulls, shifting the starting degradation point to higher temperatures (T95%). However, this parameter did not reach the value obtained for pristine cellulose, indicating the presence of low thermally stable components in the treated samples. Although the FTIR spectrum of soybean 3 is similar to that of pristine cellulose, their thermograms are different. The thermal behavior of soybean 3 can be attributed to residual non-cellulosic components, present in small amounts within the biomass, as well as to chemical modifications resulting from the treatments. As previously discussed, alkaline and acidic solutions led to the formation of new functional groups and bonds within the biomass, which may significantly influence its thermal behavior. Additionally, structural changes in cellulose—such as depolymerization—may contribute to a reduction in the thermal stability of the biomass.

Among the structural components, lignin and hemicelluloses generate considerable amounts of char, whereas lower quantities are obtained from cellulose [53]. The pyrolysis of soybean hulls produced 21.6% of char, indicating the presence of lignin and hemicelluloses in the untreated biomass. The char content was determined as the residue from pyrolysis at 700 °C minus the ash determined as the residue after heating the pyrolyzate to 800 °C in air (see Table S1).

It is worth observing that the char content in soybean 3 (14.9%) is higher than in soybean 2 (9.2%). We hypothesize that the chemical modifications occurring during the alkaline treatment favor char formation over gasification.

The combustion of soybean hulls produced a discrete amount of ash (5.3%); by contrast, ash was not detected in soybean 2 and soybean 3, indicating that the solubilization of minerals occurred. The ash of soybean hulls consists of Ca (25%), K (21%), Mg (5%), Si (1%), Al (0.8%), and Na (0.2%), in addition to C, O, P, and S. The ash of soybean 1 contains the same elements but in different percentages; among the alkali and alkaline earth metals, Ca is the most abundant element, corresponding to 37% of the ash weight, followed by Mg (8%), Na (7%), K (2%), Al (0.2%), and Si (0.2%), in addition to C, O, and P. The previous results highlighted that the chemical treatments promoted both a reduction in the ash content and a variation in its composition. As reported in the literature, several potassium-based minerals are soluble in water, whereas other solvents are required to dissolve inorganic compounds having different counterions [54]. The demineralization of biomass is promoted by diluted acidic solutions; in particular, HCl is efficient in removing Ca and Mg compounds [55]. The demineralization of soybean hulls resulted in the complete removal of inorganic compounds; the combustion of soybean 2 and soybean 3 did not generate ash.

#### 3.1.6. X-Ray Diffraction Results

**Figure 4:**
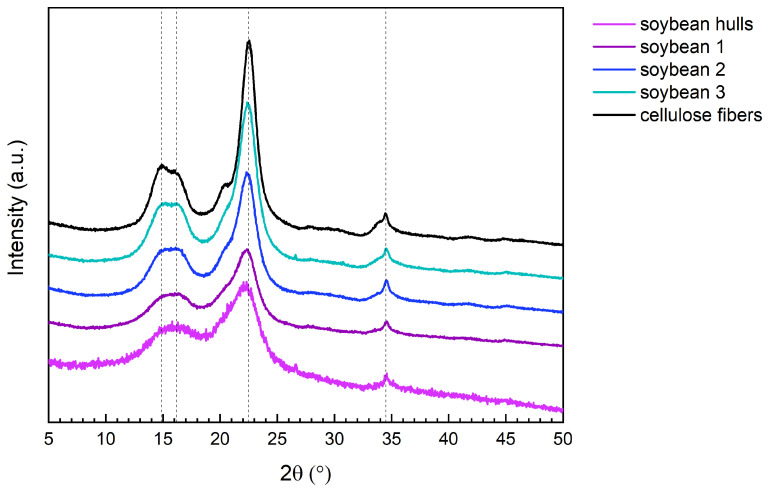
XRD of pristine cellulose and soybean hulls before and after the chemical treatments. The dashed lines correspond to the main diffraction peaks of pristine cellulose (2θ = 14.9°, 16.2°, 22.5°, 34.5°).

The diffractogram of pristine cellulose fibers (Figure 4) shows the typical signals of a type-I structure; the intense peak at 2θ = 22.5° is generated by the diffraction of the planes (200), and the less intense signals at 14.9°, 16.2°, and 34.5° correspond, respectively, to the (1–10), (110), and (004) planes [40,56].

Figure 4 and Table 3 illustrate that the type-I crystalline structure was preserved during the chemical treatments and that amorphous components were progressively removed, resulting in an increment in the crystallinity index (CI). The CI of soybean hulls (64%) increased to 74% after the first treatment, reaching 87% after the following steps. The CI of soybean 3 (87%) is just slightly lower than that of pristine cellulose (92%).

The previous results demonstrated that alkaline and acidic treatments favored the extraction of crystalline cellulose fibers. While the initial alkaline treatment induced significant alterations in the biomass composition, the subsequent steps were essential for isolating nearly pure cellulose.

### 3.2. Extraction of Cellulose from Hemp Waste

#### 3.2.1. Purification Steps and Yield Measurement

Since hemp waste can contain significantly higher amounts of lignin compared to soybean hulls [5,19], five sequential treatments were performed for cellulose extraction. Yields are presented in Table 4. The values in the table were calculated on wet weights, as we previously observed that moisture only slightly affects the yield calculation. The chemical treatments performed on hemp waste promoted the removal of non-cellulosic components, resulting in a final yield of 47%.

#### 3.2.2. FTIR Spectroscopy

**Figure 5:**
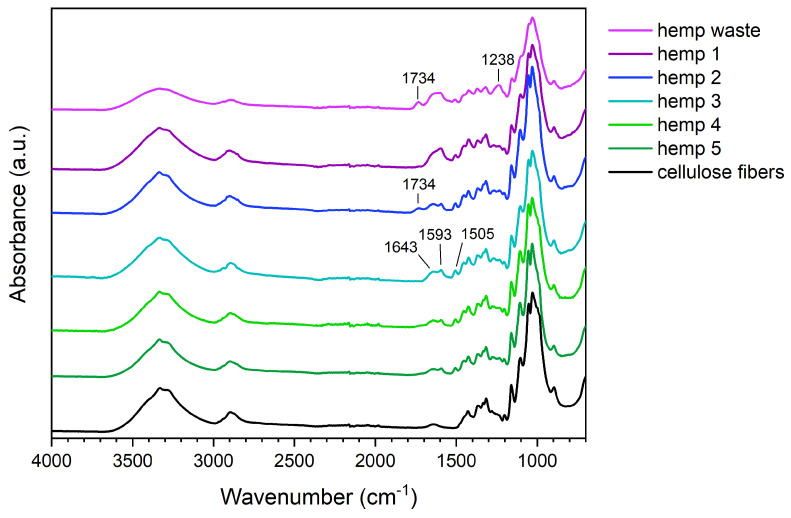
FTIR spectra of pristine cellulose and hemp waste before and after the chemical treatments.

In the FTIR spectra of hemp waste (Figure 5), three groups of signals can be detected in the region 1180–1780 cm^−1^: a peak at 1734 cm^−1^, a broad band between 1530 and 1700 cm^−1^, and a peak at 1238 cm^−1^. The intensity of these signals, generated from non-cellulosic components, was progressively reduced after the chemical treatments. For a detailed discussion of FTIR signals, refer to Section 3.1.2.

It is worth noting that the spectrum of hemp 5 still differs from pristine cellulose for the presence of two weak additional signals at 1593 cm^−1^ and 1505 cm^−1^. These signals, generated by the vibrations of aromatic rings [45], highlight that alkaline and acidic treatments did not completely remove lignin from hemp waste.

#### 3.2.3. Morphological Analyses

**Figure 6:**
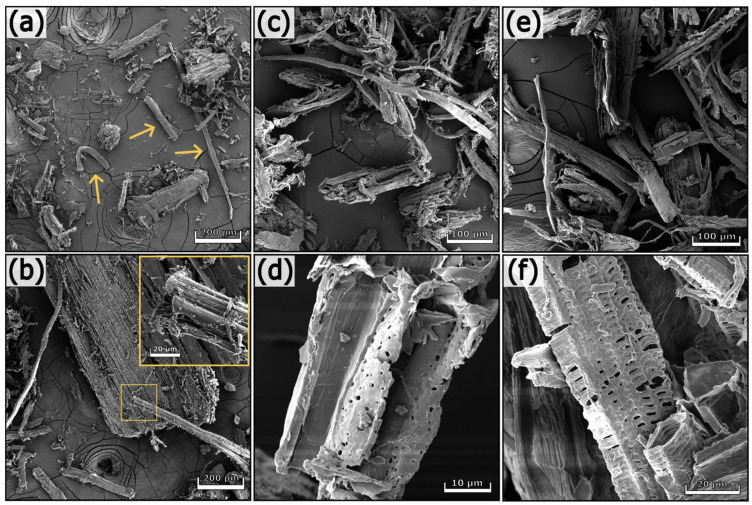
SEM images of hemp waste (**a**,**b**), hemp 1 (**c**,**d**), and hemp 2 (**e**,**f**) at different magnifications.

**Figure 7:**
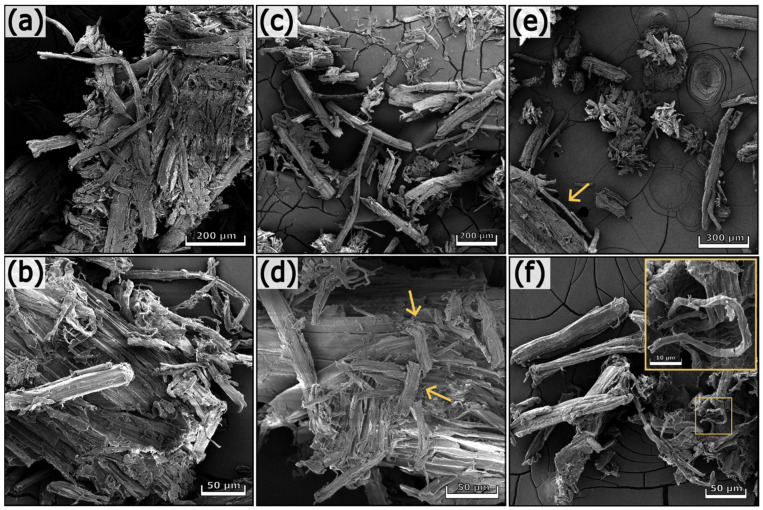
SEM images of hemp 3 (**a**,**b**), hemp 4 (**c**,**d**), and hemp 5 (**e**,**f**) at different magnifications.

The SEM pictures of untreated and treated hemp waste are presented in Figure 6 and Figure 7. Hemp waste contains cell agglomerates and individual fibers with different morphologies and dimensions (see the arrows in Figure 6a). In addition to fibers, other types of cells constitute hemp waste. Figure 6d shows elongated cells having a length of 40–45 µm and a diameter of approx. 15 µm. These cells are characteristic of xylem tissue, have lignified secondary cell walls, and contain several pores, called pits, which enable communication between cells [33]. Completely different structures from those previously described are visible in Figure 6f.

The chemical treatments progressively removed non-cellulosic components, resulting in cell disaggregation. However, the process was not complete; in fact, agglomerates can still be observed in Figure 7e (see the arrow). Hemp 5, the final product of the chemical treatment, is a heterogeneous substrate containing aggregates and elongated structures with different sizes. Short and thick fibers with a length of 40–50 µm and a diameter of approx. 11 µm are visible in Figure 7d (see the arrows). In contrast, the detail of Figure 7f shows a thin fiber 70 µm long and 2 µm thick. The fibers in hemp 5 exhibit variable and heterogeneous dimensions, with lengths up to 800 µm and diameters ranging between 2 and 60 µm.

#### 3.2.4. Chemical Composition

Elemental analysis (Table 5) indicated the presence of proteins in hemp waste. The nitrogen content, equal to 0.65% in the untreated biomass, decreased to 0.22% after the first alkaline step and stabilized at 0.17% after the second alkaline treatment.

It is worth noting that hemp 3, hemp 4, and hemp 5 exhibited similar results concerning elemental composition and FTIR spectra. Although treatments 4 and 5 removed biomass components (see Table 4), elemental analysis and FTIR spectroscopy showed some impurities still exist.

#### 3.2.5. Thermogravimetric Analyses

**Figure 8:**
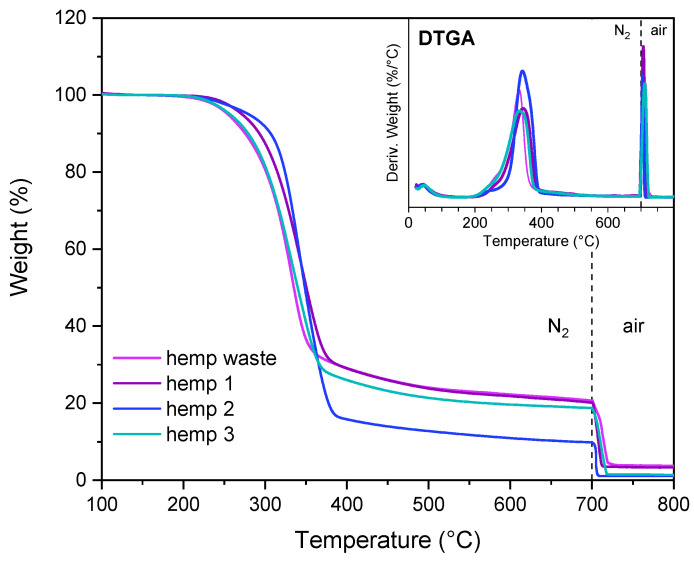
TGA and DTGA of hemp waste, hemp 1, hemp 2, and hemp 3. The dashed line represents the transition of the gas flow from nitrogen to air.

**Figure 9:**
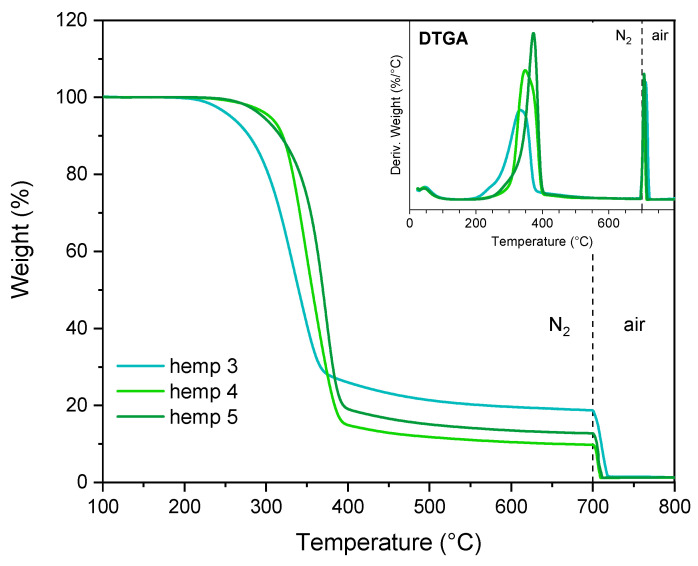
TGA and DTGA of hemp 3, hemp 4, and hemp 5. The dashed line represents the transition of the gas flow from nitrogen to air.

The results of the thermal analyses are presented in Figure 8 and Figure 9 and Table S2. The treated samples exhibited higher thermal stability than the initial biomass, indicating that less stable compounds were progressively removed. Nevertheless, the purification process was not entirely complete, as evidenced by the DTGA plots, which display a broad peak, suggesting the presence of non-cellulosic components.

The results in Table S2 show that the samples are not homogeneous; indeed, the standard deviation of the thermal parameters (T95% and DTGA peak) is one order of magnitude higher than that of pristine cellulose.

It is worth noting that the alkaline-treated biomass produced higher amounts of char than the acid-treated one. This behavior was also observed in soybean samples and confirmed that the alkaline treatment promoted chemical modifications in biomass, favoring the charring process during biomass pyrolysis.

Similar to soybean hulls, the first two steps promoted the demineralization of biomass.

#### 3.2.6. Lignin Quantification

The previous results highlighted that hemp 5 is mainly composed of cellulose; nevertheless, impurities were still detected in the sample. Since hemp waste contains various types of cells, some with lignified walls, lignin may still be present in the chemically treated biomass. To confirm the presence of lignin, even after the treatments, hemp waste and hemp 5 were subjected to cysteine-assisted acid hydrolysis (CASA method). The total lignin content determined with the CASA method corresponds to 17.7% in hemp waste and 19.5% in hemp 5. For a better interpretation of the previous results, the TAPPI protocol was applied to the biomass, and the total lignin content was calculated as the sum of Klason lignin, the acid-insoluble portion, and soluble lignin. Hemp waste was found to contain 13.7% Klason lignin and 2.1% soluble lignin, whereas hemp 5 consisted of 12.7% Klason lignin and 0.9% soluble lignin. The TAPPI method led to lower values of total lignin than the CASA procedure, regardless of the sample (see Table S3). These results suggest that other substances interfere with lignin, leading to an overestimation in the CASA method; furfural and hydroxymethyl furfural, the main acid-catalyzed degradation products of hexoses and pentoses, have their maximum absorption peak around 283 nm [57], the wavelength used for lignin quantification with the CASA method. To confirm the interference of these degradation products, we performed the CASA treatment on pristine cellulose fibers, using the same conditions previously reported. In the UV-Vis spectrum of hydrolyzed cellulose, an absorbance value of 0.16 was recorded at 283 nm. The innovative method proposed by Lu et al. [39], known as the CASA method, requires low amounts of reactants to quantify lignin. In comparison to conventional procedures (TAPPI T 222 om-02, NREL protocol [58]) that determine lignin through gravimetry and UV-Vis spectroscopy, the CASA method exploits cysteine to hydrolyze lignin and quantify it in a single stage. Even though this method shows several advantages, we observed an overestimation of lignin compared to the TAPPI protocol, indicating that the wavelength employed in the CASA method for lignin quantification should be carefully chosen to minimize the interference of the degradation products of polysaccharides. As reported by Hyman et al. [57], the signal generated by furfural is negligible at 320 nm. However, when hydrolyzing cellulose with cysteine and sulfuric acid, we still recorded an absorbance of 0.07 at 320 nm, although this was significantly lower than the value observed at 283 nm (0.16 nm). The NREL protocol [58] specifies the molar absorption coefficient at 320 nm to quantify soluble lignin within corn stover; however, the value indicated by the method may not be valid for a system containing lignocellulose components solubilized by cysteine. Given the issues identified with the CASA method, we concluded that the TAPPI protocol currently represents the most reliable approach for determining lignin content within biomass.

It is worth noting that, in the case of hemp fibers, which have a significantly higher lignin content than soybean hulls, the chemical treatments are not fully effective in promoting its removal, suggesting that additional purification treatments are required.

#### 3.2.7. X-Ray Diffraction Results

The diffractograms in Figure S4 present the typical signals of cellulose I, demonstrating that the chemical treatments preserved the crystalline structure of native cellulose. As reported in Table 6, the CI of hemp waste (79%) increased to 84% after the first treatment and remained constant in the following steps. Considering that hemp 5 contains approximately 14% lignin (see Table S3) in addition to cellulose, it is evident that the calculated CI (87%) is slightly overestimated. The results in Table 6 should be considered as an estimate of the actual crystallinity.

## 4. Perspectives and Challenges

The results presented above demonstrate that fibers rich in crystalline cellulose were successfully isolated from soybean hulls and hemp waste following sequential alkaline and acidic treatments. Although these fibers still retain a small amount of non-cellulosic components, they are potentially applicable as reinforcing agents of polymeric matrices. For instance, Martelli-Tosi et al. [59] incorporated chemically treated soybean straw into soy protein isolate and tested the tensile properties of the resulting composites. Although the chemical treatments (alkaline and bleaching) were less effective than those used in our study, yielding fibers containing 62–67% cellulose, 9–11% hemicelluloses, and 3–5% lignin, the composites with treated fibers showed higher tensile strength and a greater Young’s modulus. In contrast, adding untreated fibers did not significantly modify the mechanical performance of soy protein isolate. The authors attributed the enhancement in the mechanical properties to hydrogen bonds formed between matrix polar groups and fiber hydroxyl groups. The removal of hemicelluloses and lignin favored the formation of this network.

Similar results were obtained by Lu et al. [60], who investigated the effect of untreated and alkaline-treated hemp fibers on the mechanical performance of recycled HDPE. The incorporation of treated fibers led to a marked improvement in the tensile and flexural properties of the polymer, whereas only a slight increment was observed for the composites containing untreated fibers.

While several studies have demonstrated the reinforcing potential of cellulosic fibers extracted from hemp and soybean straw, the presence of polar functional groups on the cellulose backbone can limit their compatibility with certain polymers. To improve interfacial adhesion with hydrophobic polymer matrices and enhance the mechanical performance of the resulting composites, cellulose is often subjected to physical or chemical modifications [61]. In this context, additional treatments may be explored to tailor the surface properties of the fibers isolated in the present study and expand their applicability in the field of polymer composites. Moreover, the extracted fibers could be chemically modified to introduce new functionalities—such as antibacterial activity [50,62]—further broadening their potential applications.

## 5. Conclusions

Soybean hulls and hemp waste were chemically treated in mild conditions with NaOH 2% *w*/*v* and HCl 1 M to recover cellulose from agricultural residues. The yield of soybean hulls after three sequential treatments (two alkaline, one acid) was 35%, whereas a yield of 47% was obtained for hemp waste after five treatments (three alkaline, two acid). All the characterization techniques (FTIR, elemental analysis, TGA, SEM, EDX, XRD) highlighted that alkaline and acidic treatments promoted the removal of organic compounds and minerals, increasing the cellulose content in biomass after each step. The final product from soybean hulls (soybean 3) consists of fibers 35–50 µm long and 5–11 µm thick, containing nearly pure cellulose. The chemical treatments did not significantly affect the structure of cellulose, which consists of chains arranged in highly crystalline domains with the cellulose type-I structure. By contrast, the purification processes modified the thermal stability of biomass, enhancing its processing interval and applicability in polymer composites.

Hemp 5, the purified product of hemp waste, contains fibers of variable sizes, with diameters ranging between 2 and 60 µm and lengths from 40 to 800 µm. In addition to fibers, cell aggregates were observed in hemp 5, indicating that some substances acting as glue between adjacent cells were not completely removed. Although hemicelluloses, proteins, and other components were successfully removed, lignin showed higher resistance to alkaline and acidic treatments. The analyses revealed that a significant amount of lignin (~14%) was still present in hemp 5, indicating the need for harsh treatments to obtain nearly pure cellulose.

This study highlights that agricultural waste can be valorized by recovering cellulose fibers through alkaline and acidic treatments in mild conditions. However, while high-purity cellulose can be successfully isolated from biomass with a low lignin content, bleaching or other harsh treatments are essential to obtain nearly pure cellulose from substrates containing considerable amounts of lignin.

## Figures and Tables

**Table 1:** Yields of the chemical treatments performed on soybean hulls. The values in the second column were calculated on the initial soybean hulls, while the yields in the third and fourth columns were determined considering the weights before and after each treatment. The yields in the fourth column were calculated on the dry biomass.

	Recovered Biomass (%)	Step Yield% (Wet Biomass)	Step Yield% (Dry Biomass *)
Soybean 1	53	53	57
Soybean 2	46	86	85
Soybean 3	35	76	77

* the moisture content was determined by TGA.

**Table 2:** Composition of pristine cellulose and soybean hulls before and after the chemical treatments. The C, H, and N content are expressed as wt% of the wet samples.

	N (%)	C (%)	H (%)
soybean hulls	1.60 ± 0.03 ^a^	40.58 ± 0.12 ^a^	5.95 ± 0.03 ^a^
soybean 1	0.18 ± 0.01 ^b^	40.89 ± 0.09 ^b^	6.07 ± 0.05 ^b^
soybean 2	0.16 ± 0.01 ^b^	42.45 ± 0.09 ^c^	6.18 ± 0.02 ^c^
soybean 3	0.16 ± 0.04 ^b^	42.13 ± 0.12 ^d^	6.13 ± 0.01 ^b^
cellulose fibers	-	42.86 ± 0.08 ^e^	6.27 ± 0.07 ^c^

^a–e^ Averages with different superscript letters in the same column are statistically different (*p* < 0.05).

**Table 3:** Crystallinity index (CI) of pristine cellulose and soybean hulls before and after the chemical treatments.

	CI (%)
soybean hulls	64
soybean 1	74
soybean 2	84
soybean 3	87
cellulose fibers	92

**Table 4:** Yields of the chemical treatments performed on hemp waste. The values in the second column were calculated on the initial hemp waste, while the yields in the third column were determined considering the weights before and after each treatment.

	Recovered Biomass (%)	Step Yield%
Hemp 1	75	75
Hemp 2	67	88
Hemp 3	57	86
Hemp 4	53	92
Hemp 5	47	88

**Table 5:** Composition of hemp waste before and after the chemical treatments. The C, H, and N content are expressed as wt% of the wet samples.

	N (%)	C (%)	H (%)
hemp waste	0.65 ± 0.01 ^a^	43.11 ± 0.03 ^a^	5.92 ± 0.13 ^a,b^
hemp 1	0.22 ± 0.01 ^b^	42.73 ± 0.12 ^b^	5.82 ± 0.03 ^a^
hemp 2	0.21 ± 0.01 ^b^	44.22 ± 0.14 ^c^	5.95 ± 0.04 ^b^
hemp 3	0.17 ± 0.01 ^c^	43.91 ± 0.08 ^d^	5.98 ± 0.07 ^b^
hemp 4	0.18 ± 0.01 ^c^	44.35 ± 0.14 ^c,e^	5.95 ± 0.07 ^b^
hemp 5	0.17 ± 0.01 ^c^	44.48 ± 0.07 ^e^	5.95 ± 0.05 ^b^

^a–e^ Averages with different superscript letters in the same column are statistically different (*p* < 0.95).

**Table 6:** Crystallinity index (CI) of hemp waste before and after the chemical treatments.

	CI (%)
hemp waste	79
hemp 1	84
hemp 2	86
hemp 3	86
hemp 4	87
hemp 5	87

## Data Availability

The original contributions presented in the study are included in the article/supplementary material, further inquiries can be directed to the corresponding author.

## Supplementary Materials

The following supporting information can be downloaded at https://www.mdpi.com/article/10.3390/polym17091220/s1: Figure S1: Soybean hulls after alkaline (soybean 1, soybean 3) and acidic (soybean 2) treatments; Figure S2: SEM image of soybean hulls. Palisade cells (PL), hourglass cells (HG), and parenchyma tissue (PA) form the hull structure; Figure S3: TGA and DTGA of pristine cellulose and soybean hulls; Figure S4: XRD of pristine cellulose and hemp waste before and after the chemical treatments. The dashed lines correspond to the diffraction peaks of pristine cellulose (2θ= 14.9°, 16.2°, 22.5°, 34.5°); Table S1: Parameters obtained by TGA and DTGA of pristine cellulose and soybean hulls before and after the chemical treatments; Table S2: Parameters obtained by TGA and DTGA of hemp waste before and after the chemical treatments; Table S3: Amounts of lignin in hemp waste and hemp 5, determined by TAPPI and CASA methods.
