# Supplementary material for: Cellulose Extraction from Soybean Hulls and Hemp Waste by Alkaline and Acidic Treatments: An In-Depth Investigation on the Effects of the Chemical Treatments on Biomass
Source: Polymers (Basel). 2025 Apr 29;17(9):1220. doi: 10.3390/polym17091220 (PMC12073554; doi:10.3390/polym17091220)
Supplement: Supplementary file 1 [file polymers-17-01220-s001.zip › polymers-3598182-supplementary.pdf]

Supplementary material

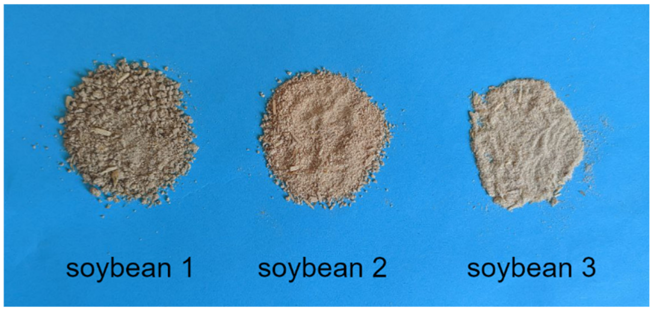

Figure S1: Soybean hulls after alkaline (soybean 1, soybean 3) and acidic (soybean 2) treatments.

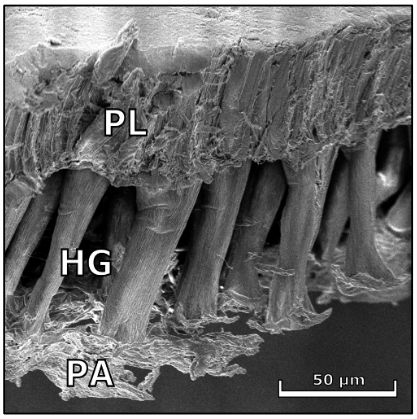

Figure S2: SEM image of soybean hulls. Palisade cells (PL), hourglass cells (HG), and parenchyma tissue (PA) form the hull structure.

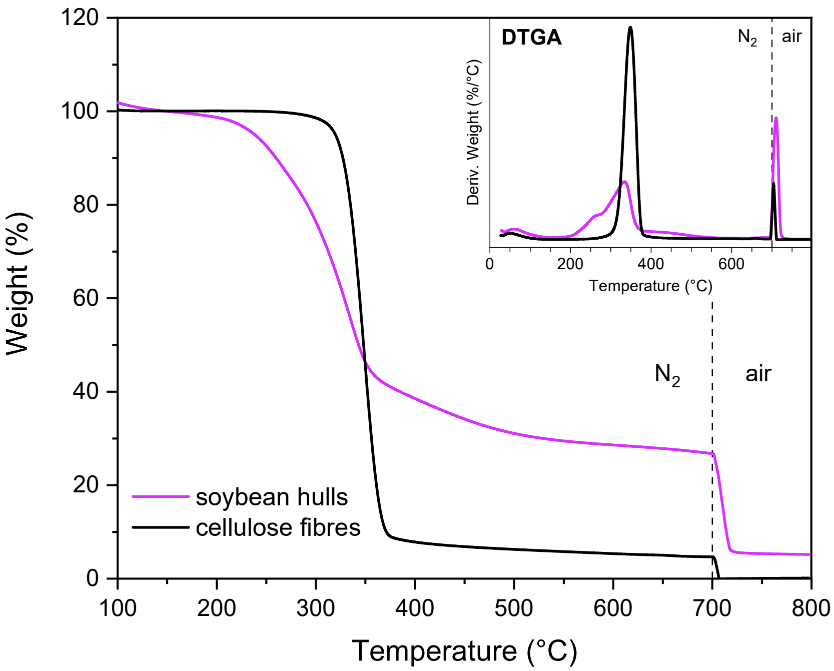

Figure S3: TGA and DTGA of pristine cellulose and soybean hulls.

Table S1: Parameters obtained by TGA and DTGA of pristine cellulose and soybean hulls before and after the chemical treatments.

	T 95% (°C)	DTGA peak (°C)	Residue 700°C (%)	Ash (%)
soybean hulls	238.5±0.3	336±3	26.9±0.2	5.3±0.1
soybean 1	267±4	354.0±0.5	23.7±0.3	3.3±1.1
soybean 2	266±2	334.2±1.0	10.5±0.1	-
soybean 3	271.8±0.8	355.8±1.0	16.4±1.3	-
cellulose fibers	319.8±0.4	348.6±0.1	5.1±0.6	-

Table S2: Parameters obtained by TGA and DTGA of hemp waste before and after the chemical treatments.

	T 95% (°C)	DTGA peak (°C)	Residue 700 °C (%)	Ash (%)
hemp waste	249±5	330±4	21.0±0.6	3.9±0.2
hemp 1	265±6	339±7	20.1±0.1	2.9±0.5
hemp 2	281±3	344±2	10.1±0.4	-
hemp 3	259±3	341±8	18.6±0.1	-
hemp 4	304.5±0.3	348.8±0.6	9.4±0.5	-
hemp 5	293±5	370±4	12.5±0.3	-
cellulose fibers	319.8±0.4	348.6±0.1	5.1±0.6	-

Table S3: Amounts of lignin in hemp waste and hemp 5, determined by TAPPI and CASA methods.

	% total lignin (TAPPI)	% total lignin (CASA)
hemp waste	15.8	17.7
hemp 5	13.6	19.5

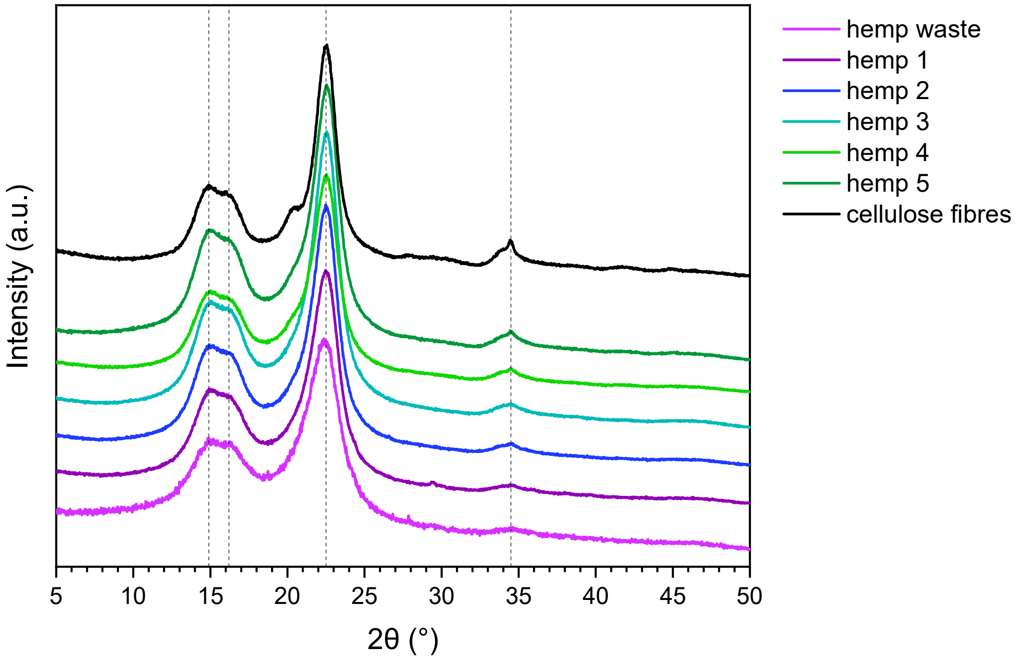

Figure S4: XRD of pristine cellulose and hemp waste before and after the chemical treatments. The dashed lines correspond to the diffraction peaks of pristine cellulose (2θ= 14.9°, 16.2°, 22.5°, 34.5°).
